# Incidence of Vertical Phoria on Postural Control During Binocular Vision: What Perspective for Prevention to Nonspecific Chronic Pain Management?

**Published:** 2015

**Authors:** Eric Matheron, Zoï Kapoula

**Affiliations:** Groupe IRIS, Physiopathologie de la Vision et Motricité Binoculaire. CNRS FR3636, Neurosciences, UFR Biomédicale, Université Paris Descartes, 45, rue des Saints-Pères, 75006 Paris, France

**Keywords:** Vision, Vertical Heterophoria, Postural Control, Back Pain, Nonspecific Pain, Sensorimotor Conflict, Prevention

## Abstract

Vertical heterophoria (VH) is the latent vertical misalignment of the eyes when the retinal images are dissociated, vertical orthophoria (VO) when there is no misalignment. Studies on postural control, during binocular vision in upright stance, reported that healthy subjects with small VH vs. VO are less stable, but the experimental cancellation of VH with an appropriate prism improves postural stability. The same behavior was recorded in nonspecific chronic back pain subjects, all with VH. It was hypothesized that, without refraction problems, VH indicates a perturbation of the somaesthetic cues required in the sensorimotor loops involved in postural control and the capacity of the CNS to optimally integrate these cues, suggesting prevention possibilities. Sensorimotor conflict can induce pain and modify sensory perception in some healthy subjects; some nonspecific pain or chronic pain could result from such prolonged conflict in which VH could be a sign, with new theoretical and clinical implications.

Today, it is not possible to ignore the role of vision and eye movements involved in postural control and the tonic activity in Human (eg (1-8)). Vision and gaze signals influence the vestibuloocular, the vestibulospinal and the reticulospinal systems (see (9)). And we cannot ignore the importance of body somatosensory signals on the mobilization of the eye and the visual perception of our surrounding space (eg (10-15)). In the daily life, balance, posture and movement control is complex. For instance, postural control in quiet upright stance, the Human reference posture and also the basis for body stability during movements and gait (see (16,17)), requires the central integration of visual, vestibular and somaesthetic inputs. The central nervous system performs appropriate coordinate transformations of these inputs and permanently generates adapted muscular response (e.g.(4,18)). 

 Various experimental investigations were run to contribute for better understanding about the mechanisms and complexes interaction between vision, oculomotricity, and postural control, notably studies about vertical phoria. When the retinal images are dissociated, vertical heterophoria (VH) is the latent vertical misalignment of the eyes reduced via binocular vision mechanisms, and vertical orthophoria (VO) when there is no misalignment (19,20). Physiological VH was reported in normal subjects, inferior to one diopter, on average 0.28 diopter, corresponding to 0.16±0.01° (21). Nevertheless, studies reported that VH experimentally induced in healthy young adult subjects by the insertion of a 1 degree-vertical prism influences postural stability, notably a decrease of postural stability during the quiet upright stance (22). Other results clearly showed that healthy subjects with VH vs. VO (VH less than 1 diopter) are less stable, but the cancellation of VH with an appropriate prism improves postural stability (23). In line with this, in nonspecific chronic back pain subjects with an additional comorbidity such as peripheral arthralgia, muscle pain, dizziness, headache, or eyestrain known (e.g. (24,25)), representing up to 85% of back pain (26), all with VH, the same behavior was recorded (27). Interestingly, these subjects needed more energy to stabilize their posture compared in healthy subjects, but the energetic cost decreased when VH was canceled (27). Beside eye refraction problems (19,28), it was hypothesized that VH, even when small in size, indicates a perturbation of the somaesthetic cues required in the sensorimotor loops involved in postural control and could perhaps indicate the capacity of the central nervous system to integrate these cues optimally, suggesting prevention possibilities (see (23)). 

 It is known that sensorimotor conflict (at least between vision and proprioceptive cues) can induce pain and modify sensory perception in some healthy subjects (29); we hypothesized that non-specific chronic back pain could result from such prolonged conflict in which VH could be a sign, with new theoretical and clinical implications (see (27)). This speculation, beyond to be in line with the experimental model introduced by McCabe et al. (29), agrees with the suggestions of Harris (30): i) discordance between awareness of motor intention, muscle and joint proprioception, and vision could lead to pain perception, and ii) when pain exists – without medical pathologic support as organic lesion, rheumatism, neuropathy nor injuries, sprain or fracture –, instead of treating the painful body part with medication, the therapy management should “ be directed at restoring the integrity of cortical information processing”. 

 Clinical studies suggested the use of VH detection - via the Maddox Rod Test, one of the more robust for the small vertical deviation (31,32) - as a landmark in the management of nonspecific chronic pain, non-contact injuries (e.g. tendinitis, muscle pain). For instance, VH can be linked to conflict from the stomatognathic system, the pelvis, piercings (body art, i.e., with jewelry) or refractive error; cancel the conflict most of the time restored VO immediately, diminished pain (33-35), improved mobility of spinal and peripheral joints, and normalized behaviour in the balance tests after initial alternation (33,34,36), but remain to be precisely evaluated – e.g. with objective measurements of heterophoria (e.g. with the use of an eye tracker) and movement (e.g. 3D motion capture). The exact causal relationship has to be identified. Nevertheless, as previously discussed on theoretical aspects (35), these various afferences (i.e. visual, vestibular and somaesthetic) project to the cerebellum, the reticular formation, and the vestibular nucleus (see (37)) which are located at the base of the spinal motor neurons and oculomotor efferents (see (38)). Yet, based on these experimental studies and clinical reports, on the impact of afferences required for motor control in various tasks and muscle efficiency performance, the possibility of degradation of postural control in upright stance and possible pain linked to sensorimotor conflict, perspective in optimization of muscular efficiency and at least in nonspecific chronic pain management should be of interest.
